# Effects of Sport Stacking on Neuropsychological, Neurobiological, and Brain Function Performances in Patients With Mild Alzheimer's Disease and Mild Cognitive Impairment: A Randomized Controlled Trial

**DOI:** 10.3389/fnagi.2022.910261

**Published:** 2022-05-12

**Authors:** Ziying Yang, Wenbo Zhang, Dunxiu Liu, Shan-shan Zhang, Yong Tang, Jiaqi Song, Jinfeng Long, Jun Yang, Hong Jiang, Yaling Li, Xintong Liu, Yang Lü, Fu Ding

**Affiliations:** ^1^Department of Nursing, The First Affiliated Hospital of Chongqing Medical University, Chongqing, China; ^2^Department of Geriatrics, The First Affiliated Hospital of Chongqing Medical University, Chongqing, China; ^3^Department of General Practice, The First Affiliated Hospital of Chongqing Medical University, Chongqing, China; ^4^Department of Histology and Embryology, Faculty of Basic Medical Sciences, Chongqing Medical University, Chongqing, China; ^5^Laboratory of Stem Cell and Tissue Engineering, Chongqing Medical University, Chongqing, China; ^6^Institute of Neuroscience, Chongqing Medical University, Chongqing, China

**Keywords:** sport stacking, AD, MCI, Alzheimer's disease, mild cognitive impairment, neuropsychological, neurobiological, fNIRS

## Abstract

**Objective:**

To investigate the effects of sport stacking on the overall cognition and brain function in patients with mild Alzheimer's disease (AD) and mild cognitive impairment (MCI).

**Methods:**

A single-blind randomized controlled design was performed using sport stacking for 30 min, 5 days/week for 12 weeks. Forty-eight subjects with mild AD or MCI were randomly divided into the sport stacking group (T-mAD = 12, T-MCI = 12) and the active control group (C-mAD = 11, C-MCI = 13). Auditory Verbal Learning Test (AVLT), Alzheimer's Disease Cooperative Study–Activities of Daily Living scale (ADCS-ADL), Geriatric Depression Scale (GDS-30), and Pittsburgh Sleep Quality Index (PSQI) were performed, the level of amyloid β-protein-40 (Aβ-40), Aβ-42, brain-derived neurotrophic factor (BDNF), insulin-like growth factor-1(IGF-1), tumor necrosis factor-alpha (TNF-α), Interleukin-6 (IL-6), and soluble trigger receptor expressed on myeloid cells 2 (sTREM2) in plasma were tested, and brain functional connectivity in resting state and activation under finger movement task were analyzed by functional near-infrared spectroscopy (fNIRS).

**Results:**

Thirty-nine patients completed the trial. After 4 weeks, we found a significant increase in AVLT score in T-MCI (6.36 ± 5.08 vs. −1.11 ± 4.23, *p* = 0.004), and T-mAD group (4.60 ± 4.77 vs. −0.11 ± 2.89, *p* = 0.039). After 12 weeks, there was a significantly improved in AVLT (9.64 ± 4.90 vs. −0.33 ± 6.10, *p* = 0.002) and ADCS-ADL (3.36 ± 3.59 vs. −1.89 ± 2.71, *p* = 0.003) in T-MCI. There was a significant improvement in AVLT (5.30 ± 5.42 vs. 0.44 ± 2.40) in T-mAD (*p* < 0.05). Plasma levels of BDNF were upregulated in both T-MCI and T-mAD, and IGF-1 increased in T-MCI (*P* < 0.05) compared to the control groups. The functional connectivity in MCI patients between DLPFC.R and SCA.R, SMA.L, and SCA.R was decreased. In contrast, in mAD patients, the brain regional function connection was increased between DLPFC.R and Broca's.L. The activation of channel 36 located in the left primary somatosensory cortex was significantly increased after 12-week training, which was correlated with the improved AVLT and the increase of BDNF.

**Conclusion:**

Our findings suggested that sport stacking is effective for patients with MCI and mild AD, possibly through increasing the expression of neuroprotective growth factors and enhancing neural plasticity to improve neurocognitive performance.

**Clinical Trial Registration:**

https://www.ClinicalTrials.gov, ChiCTR.org.cn, identifier: ChiCTR-2100045980.

## Introduction

Mild cognitive impairment (MCI) is an intermediate state between normal aging and dementia, with about 15% would progress to dementia in 2 years (Petersen et al., 2018), and one-third (32%) develop Alzheimer's disease (AD) within 5 years (Ward et al., 2013). As predicted, the number of people with dementia will reach to 78 million by 2030 (Gauthier et al., 2021). However, there is still a lack of effective pharmacological treatment today for AD, the most common cause of dementia. In recent years, non-pharmacologic treatments have drawn wide attention by showing their significant role in improving and maintaining cognitive function, quality of life, and daily function in patients with different severity of cognitive impairment. Moreover, a recent review found non-pharmacologic treatments seemed to be more effective than pharmacologic treatments for agitation and aggression in people with dementia (Watt et al., 2019). Aerobic exercise, the mainly studied physical activity among non-pharmacological treatments, has been shown to enhance the cognitive function of people with AD, reduce the risk of AD and other dementia, and delay the onset or progression (Groot et al., 2016; Hamer et al., 2018; Song et al., 2018). However, regular aerobic exercise may be difficult for dementia patients to adhere (Yágüez et al., 2011) for poor interest (Maltais et al., 2019) and low participation of the elderly (Padala et al., 2017).

Sport stacking is a new sport that began in the early 1980s. Participants use 12 specialized cups with both hands to make a pyramid (“up stacking”) and then return the cups into stacks (“down stacking”). The whole process must be in predetermined sequences (Hart et al., 2005). Sport stacking could be seen more suitable for patients with cognitive impairment because it is combined with the game and physical activity, which can trigger a high willingness to participate (Park, 2017). Previous studies showed that sport stacking was beneficial in many aspects, such as hand-eye coordination (Hart et al., 2006), reaction time (Liggins et al., 2007), bilateral coordination (Rhea et al., 2006), and dual hemispheric brain activity (Hart and Bixby, 2005). Some studies have applied sport stacking to stroke patients and found significant improvement in reaction time (Tretriluxana et al., 2014). Despite its beneficial role for cognitive improvements in other diseases, there is still a lack of evidence for the effect of sport stacking on people with dementia.

Growing evidence suggests that AD is a multifactorial disease that affects the central nervous system and systemic processes (Morris et al., 2014). More specifically, increased inflammation and production of reactive oxygen species (ROS) might also play an essential role in the pathophysiology of MCI and AD (Gilgun-Sherki et al., 2001; Rosenberg, 2005; Koyama et al., 2013). Evidence shows that physical activity appears to have a positive effect on inflammation, oxidation, and neurotrophic biomarkers by enhancing the antioxidant activity of plasma and reducing the serum expression of proinflammatory cytokines, which may affect the destructive effects of oxidative stress and inflammation in nerve tissue (Stigger et al., 2019). Physical exercise has been shown to produce an increase in brain-derived neurotrophic factor (BDNF) and variable response to insulin-like growth factor-1 (IGF-1) (Anderson-Hanley et al., 2018). However, the evidence for the effect of sport stacking on these biomarkers as well as AD biomarkers is unknown.

Functional Near-Infrared Spectroscopy (fNIRS) is a new brain mechanism functional imaging technology which can perform advanced cognition and interactive behavior in natural situations. It makes up for the limitations of detection tools such as single-photon emission computed tomography (SPECT), positron emission tomography (PET), and functional magnetic resonance imaging (fMRI) (Liu et al., 2011; Yeung and Chan, 2020). fNIRS can evaluate the activation of different brain regions by observing the changes of oxygenated hemoglobin (Oxy-Hb), deoxyhemoglobin (Deoxy-Hb), and total hemoglobin (Total-Hb) concentration curves in different brain regions during the cognitive process. Hoshi (2011) indicated that monitoring the changing trend of blood oxygen concentration in the prefrontal cortex while completing cognitive tasks could objectively reflect the subjects' cognitive level. Since the abilities of language comprehension, execution of an action, working memory, and movements are needed in fulfilling sport stacking, the brain regions of interest (ROI) were selected in corresponding to these functions. Recent studies have shown that the dorsolateral prefrontal cortex (DLPFC) is a crucial area for processing various behavioral tasks, and specifically, the right DLPFC (DLPRC.R) modulates the direction of these tasks (Xia et al., 2021) and semantic cognition (Herbet et al., 2018). Broca's area, a prefrontal region that was demonstrated to not only be involved in language production and comprehension but also play a role in several non-language-related functions such as working memory, execution, and perception of action (Clos et al., 2013; Kepinska et al., 2018). In addition, the subcentral area (SCA), also known as the subcentral motor cortex, and left supplementary motor area (SMA.L) are correlated with hand movements and grip (White et al., 2013; Auer et al., 2018; Eichert et al., 2021). Therefore, we select DLPFC.R, SCA.R, SMA.L, and Broca's area as the ROIs in this study.

This study aimed to investigate the effects of sport stacking on the neurocognitive performances, molecular biomarkers, and brain function performances in patients with mild AD and MCI. We hypothesized that sport stacking would effectively improve the cognitive function of patients with mild AD and MCI, potentially through divergent molecular factors (e.g., neuroprotective growth factors and cytokines) and the changes in activation of brain areas examined by fNIRS.

## Materials and Methods

### Study Design

The current study was designed as a single-blind randomized controlled trial (RCT) of 12 weeks of sport stacking vs. a non-exercise and clinic routine management control group. Participants were included from May 2021 to September 2021. The Medical Ethics Committee of the First Affiliated Hospital of Chongqing Medical University approved the research protocol. The study was conducted in compliance with the Declaration of Helsinki's ethical standards (World Medical Association, 2013). It was registered on the Chinese Clinical Trial Registry (Registration No.: ChiCTR2100045980). Participants all agreed to participate in the study and gave written informed consent.

### Participants

Eligible elderly participants with a clinical diagnosis of dementia (DSM-IV) or NINCDS-ADRDA Alzheimer's Criteria would be recruited from the geriatric memory clinic at the First Affiliated Hospital of Chongqing Medical University between May 2021 and September 2021. Eligibility criteria for inclusion were: 1) between 60 and 90 years of age; 2) a Clinical Dementia Rating (CDR) score of 0.5 or 1; 3) at least 3 months of stable doses if receiving antidementia medication or mood-stabilizing medication; 4) basic communication skills and normal vision and hearing; and 5) informed consent. Exclusion criteria were: 1) severe psychiatric illness and the use of antidepressants; 2) alcohol or drug abuse; 3) participation in exercise more than twice weekly on a regular basis; and 4) any medical condition precluding participation in the exercise program (e.g., severe cardiovascular, musculoskeletal, or neurological disease).

### Sample Size

Cognition (ADAS-Cog) was the primary outcome measure. We calculated that a sample size of 40 participants would provide 80% power (at a two-tailed α-level of 0.05) for detecting differences between groups for an effect size of 0.46 in the ADAS-Cog (Orrell et al., 2012). Assuming a 20% attrition rate, the study recruited a total of 48 participants (24 per group). Sample size calculation was performed using G^*^Power 3.1.9 (Faul et al., 2007).

### Randomization and Blind

Forty-eight patients who met the inclusion criteria were randomized 1:1 ratio to receive either the intervention (sport stacking) or control (clinic routine management) *via* a computer-generated randomization sequence by a statistician. Allocation concealment was ensured since the randomization was performed by a research assistant who was not involved in the assessment or intervention. This was a single-blind study in which study participants were blinded to the group allocation.

### Procedure

In the sport stacking group, participants and their caregivers were taught how to stack the cups in a specific sequence with the correct technique using the lesson plans recommended in the Speed Stacks® instructor guide (Speed Stacks, 2014) by an experienced instructor at the geriatric clinic or online from baseline to the 3rd month.

Participants needed a set of stacking tools to practice, including 12 cups, a timer, and a stacking mat. To facilitate the self-practice at home, a set of audiovisual videos showing the skills trained in all lessons was provided as a reminder to guide the participants' self-practice. The whole training lessons consist of 3 patterns, including 3–3–3, 3–6–3, and the Cycle. Because of the characteristics of elderly participants with dementia, three lessons were divided into 7 stages, including 3, 3–3–3, 6, 3–6–3, 3–6–3 & 6–6, 1–10–1, and the Cycle. The difficulty of the stages increased per level to ensure that the training remained cognitively challenging. Participants were asked to practice sport stacking at least 30 min a day and at least 5 days a week for a total of 3 months at home. Meanwhile, the participants were asked to record each time they finished sport stacking and the duration of their daily self-practice at home. The caregivers were encouraged to assist the participants in the practice and compliance recording process. The participants or their caregivers were asked to video-tape the participants' self-practice on the first and last day of each week to the researcher for collecting feedback. These videos could be used to check the participants' mastery of the sport stacking mode and correct their inaccuracies promptly. Adherence to the intervention was calculated by the number of pages of the self-recording logbook and the videos of participants' self-practice.

In the control group, all participants (MCI and mAD) received routine management from the Memory Clinic, mainly including (1) Regular medication; (2) Basic health education (medication, diet, exercise, etc.).

### Experimental Procedure and Measurements

The neuropsychological evaluations were administrated at three timepoints, namely baseline before randomization, immediately after 4 weeks and after 12 weeks of sport stacking training. Blood tests and fNIRS measurements were conducted at baseline and 12 weeks. The assessments were carried out at the Memory Clinic, Department of Geriatrics, The First Affiliated Hospital of Chongqing Medical University. Under certain special circumstances, the neuropsychological evaluations of some of the participants were carried out online. And trained research assistants were blind to group allocation.

### Neuropsychological Evaluation

The Mini-mental State Examination (MMSE; Folstein et al., 1975), and the Alzheimer's Disease Assessment Scale-Cognitive Subscale (ADAS-Cog; Mohs et al., 1983) were used to evaluate the global cognition, behavior, and ability to manage daily life. The Auditory Verbal Learning Task (AVLT; Arnáiz et al., 2004) was used to assess their memory. We used the clock drawing test (CDT; Sunderland et al., 1989) to assess executive functions. The 30-Geriatric Depression Scale (GDS-30; Yesavage et al., 1982) was used to rate the severity of depressive symptoms. The Alzheimer's Disease Cooperative Study–Activities of Daily Living scale (ADCS-ADL; Galasko et al., 1997) was used to assess activities of daily living function of patients with AD and MCI. The Pittsburgh Sleep Quality Index (PSQI; Buysse et al., 1989) was used to measure sleep quality.

### Neurobiological Measurements

A 10-mL blood sample was obtained by a qualified nurse from the antecubital vein prior to and after the intervention. The blood samples were drawn to analyze serum amyloid β-protein (e.g., Aβ-40, Aβ-42), neuroprotective growth factors (e.g., BDNF, IGF-1), cytokines (e.g., TNF-α, IL-6), and markers of microglia activation and reactive oxygen species (e.g., sTREM2) levels. The blood samples were centrifuged at 3,000 rpm for 15 min, and serum was isolated and kept at −80°C until the next step. The levels of serum amyloid β-protein, neuroprotective growth factors, inflammatory cytokines, and plasma sTREM2 were analyzed using the Human ELISA kit (Shanghai Jianglai Industrial Limited by Share Ltd., Shanghai, China).

### fNIRS Tasks, Acquisition and Analysis

#### Resting State and Finger Movement Task

The 8-min resting state at the sitting position took place in a sound-attenuated room. During the resting state recording, the participants were required to stay still and keep their eyes closed without falling asleep.

**Figure 1:**
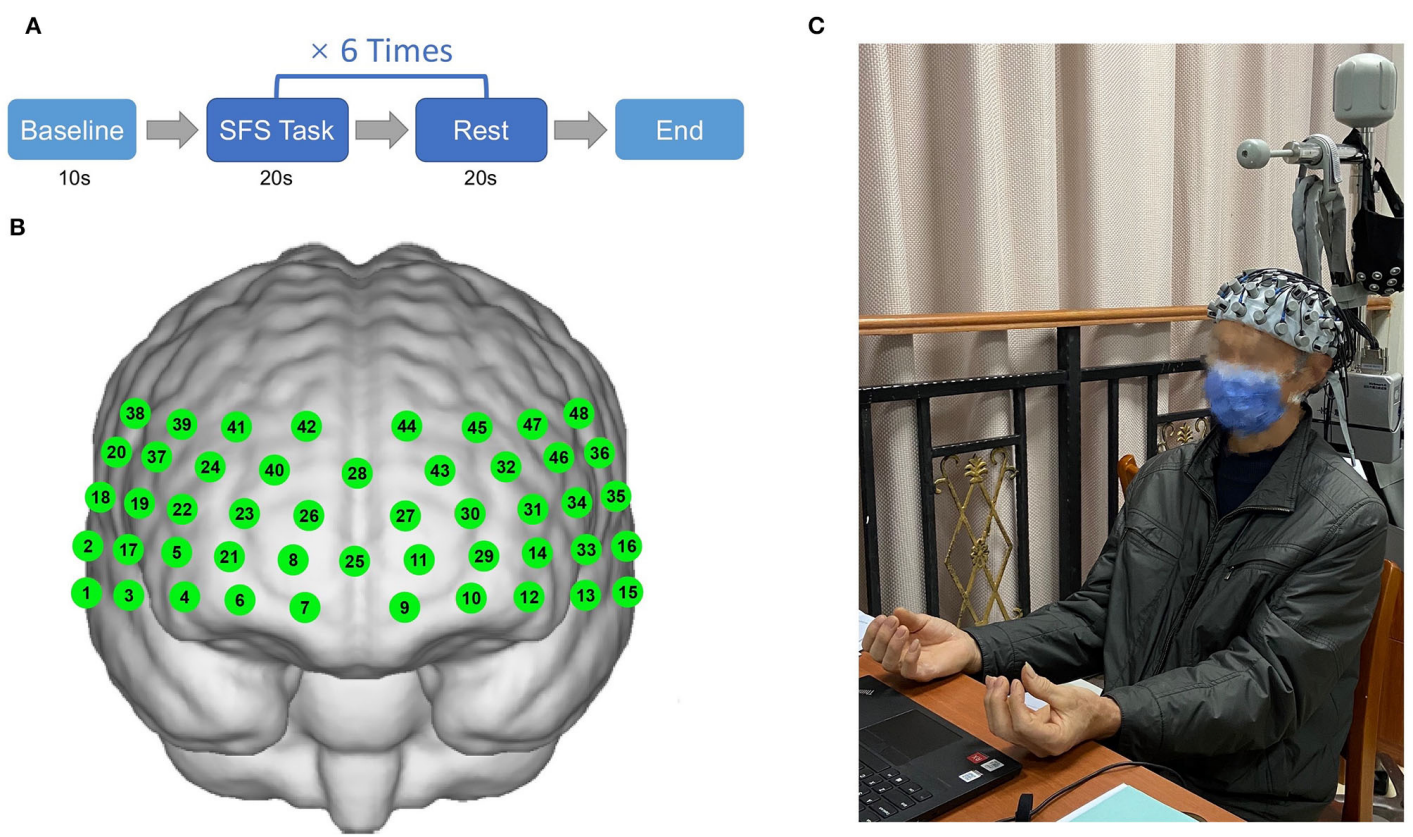
Illustration of finger movement task (simple finger sequence) paradigm **(A)** and positions of 48 channels **(B,C)** (Informed consent was obtained from the patient).

The finger movement task refers to a simple finger sequence (SFS) (Anwar et al., 2016), which requires the participants to sequentially tap the index, middle, ring, and the fourth finger against the thumb on both hands simultaneously. A block design was used in which subjects were asked to perform the finger movement task (SFS) for 20 s followed by 20 s rest for 6 repeat times (Figure 1A).

#### fNIRS Acquisition

In this experiment, the hemoglobin concentrations were measured using a multi-channel fNIRS system (NirScan, Danyang Huichuang Medical Equipment Co. Ltd, China). The sampling frequency was 11 Hz, and the wavelengths were 760 and 850 nm, according to the requirements of the internationally-used 10/20 electrode distribution system. We used the FPz channel (10/20 international system) as the center of the middle probe; a total of 31 SD probes (consisting of 15 sources and 16 detectors) with a fixed 3-cm inter-probe distance were placed to cover each subject's bilateral prefrontal cortex (PFC) and temporal cortices, and the lowest probes were positioned along with the Fp1–Fp2 line (Figures 1B,C). A total of 48 NIRS channels were established. The channels and the corresponding brain regions are consistent with a previous study (Liu et al., 2022).

#### Pre-processing

We used the NirSpark software package v1.7.3 (Danyang Huichuang Medical Equipment Co. Ltd, China) to analyze NIRS data. Data were preprocessed via the following steps. Motion artifacts were corrected by a moving SD and a cubic spline interpolation method. All differential path-length factors (DPF) were set to 6.0. According to previous studies (Li et al., 2021; Zhang et al., 2021), a bandpass filter with cut-off frequencies of 0.01–0.20 Hz was used to minimize noise, global trends, and biological signals (e.g., respiration and cardiac activity). The modified Beer-Lambert law was applied to convert optical densities into changes in Oxy-Hb and Deoxy-Hb concentrations. We used Oxy-Hb as our primary indicator in the following analysis because the Oxy-Hb signal generally has a better signal-to-noise ratio than Deoxy-Hb (Strangman et al., 2002).

#### Block Average

SFS block waveforms were calculated with a pre-baseline range set of 0–10 s and a block range set of 0–40. We used a 20 s task period of constructing phrases as the time window to analyze mean Oxy-Hb during the task and compare 20 s task period with 20 s rest period to analyze the Oxy-Hb change between task and baseline.

#### GLM Analysis

The generalized linear model (GLM) was used to analyze the Oxy-Hb time series data. The *t*-test was performed on the baseline and task state signals for each channel of each subject. The canonical hemodynamic response function (HRF) with time and dispersion derivative was selected as the basic function of the GLM. Through calculating the match between the experimental HRF value and the designs, the GLM can derive a value of activation coefficient β value representing the intensity of activation triggered by the task in the subject's cerebral cortex.

#### Functional Connectivity Analysis

The regions of interest (ROI) were selected as right dorsolateral prefrontal cortex (DLPFC.R) (channel 26, 28, and 40), left Broca's Area (Broca's. L) (channel 30, 31, and 32), left supplementary motor area cortex (SMA.L) (channel 46), and right subcentral area (SCA.R) (channel 17). For each resting-state dataset, functional connectivity (FC) was analyzed by performing Spearman's correlation between the time series of each channel-to-channel pair, resulting in a 48 × 48 matrix of R-values (Supplementary Figure 1).

#### Statistical Analysis

All statistical analyses were performed by IBM SPSS ver. 26.0 (IBM Corp., Armonk, USA). The NirSpark software package v1.7.3, GraphPad Prism 9, and Photoshop software were used to generate figures and graphs. The Shapiro–Wilk test and the Levene test were used to determine the normality and homoscedasticity of the data. All data expressed as mean ± standard deviation (SD). All the (outcome) variables were analyzed for differences between control and intervention groups using independent-sample Student's *t*-tests and the Mann–Whitney *U*-test for continuous variables, and Fisher's exact tests for categorical variables. The between-group difference in the change of score of the continuous variables from baseline to follow-up at 4 and 12 weeks were analyzed by using delta(Δ)-linear mixed models (Δ = change from baseline), where “change from baseline” represents the difference between the baseline and follow-up groups for each diagnosis, after adjusted for the premeasurement covariates (age and sex) for primary and secondary endpoint outcomes. Unpaired *t*-tests were used to compare mean and Oxy-Hb changes between mild AD and MCI cohorts, and paired *t*-tests were used to compare the *R*-values of FC and β-value of task activation between baseline and 12-week follow-up in T-mAD and T-MCI group. A *p* < 0.05 was considered statistically significant, and all *p*-values were two-tailed. The statistical results were corrected for multiple comparisons across channels by the false discovery rate (FDR). Additionally, we calculated the Pearson correlation coefficient between changes in brain region activation (β-value), BDNF levels, and neuropsychological performances (AVLT) in the T-mAD group.

## Results

A total of 48 patients participated in the study, and 39 patients completed the study (21 in the intervention group and 18 in the control group). During this period, 39 patients completed neuropsychological assessments, 32 patients gave blood samples, and 29 completed measurements of fNIRS at baseline and 12-week follow-up (mAD = 15, MCI = 1 4 ).

### Characteristics of the Study Population

**Figure 2:**
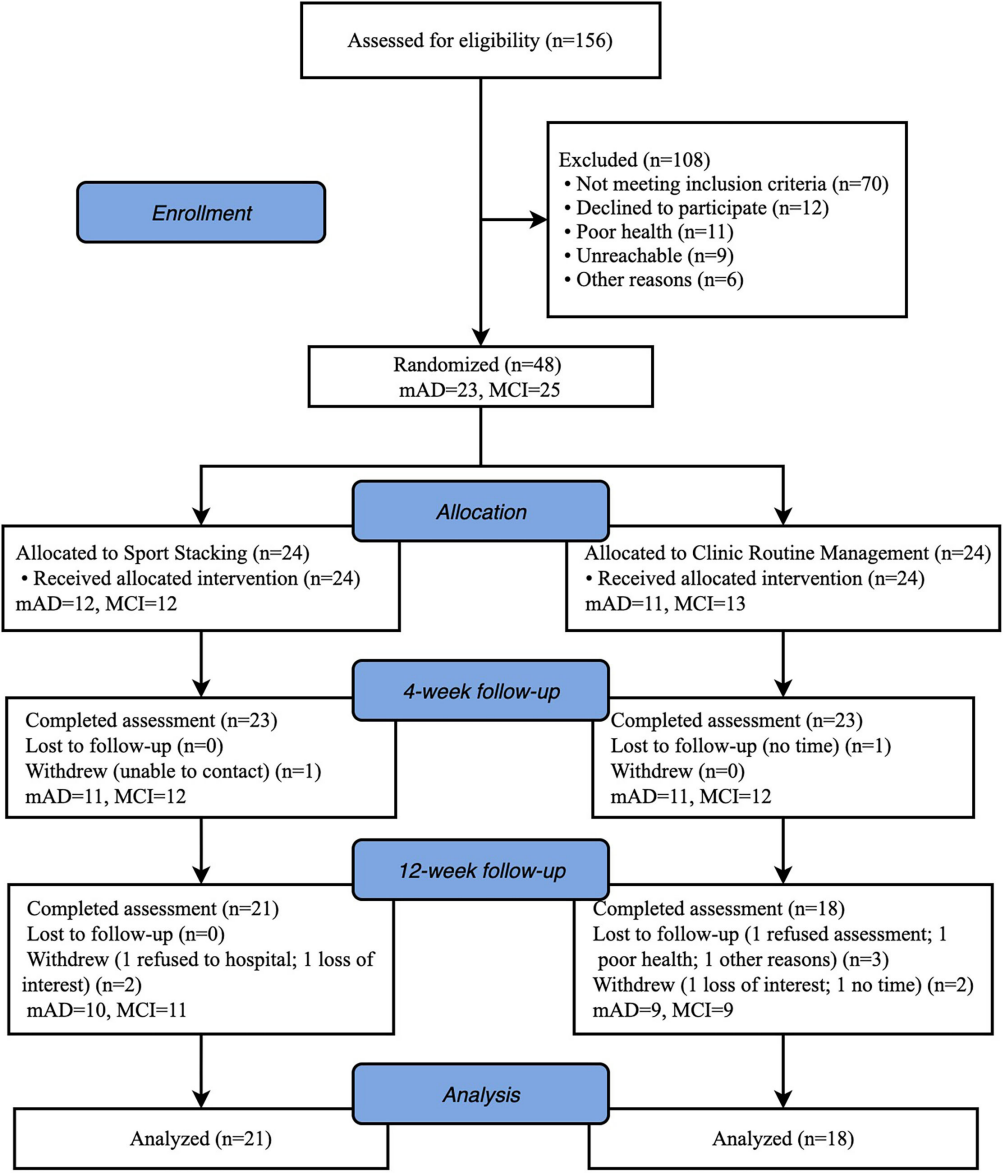
Study flow chart.

A total of 48 eligible patients, 23 with MCI and 25 with mild AD, were enrolled in the study. Twenty-four were assigned to the intervention group (IG) and 24 to the control group (CG). Before the end of the trial, 9 participants withdrew (2 loss of interest; 2 refused to hospital; 2 no time; 1 poor health; 1 unable to contact and 1 other reasons), resulting in a drop-out rate of 18.8%. Thirty-Nine patients [26 female (66.7%); mean (SD) age, 73 (6.9) years] completed the study at last (IG: *n* = 21; CG: *n* = 18). The Consolidated Standards of Reporting Trials (CONSORT) flowchart outlining the number of participants from screening to study completion at 12-week follow-up is shown in Figure 2. There were no significant statistical differences in characteristics or neuropsychological assessment results between the groups at baseline. Baseline characteristics of the study population are shown in Table 1.

**Table 1:** Baseline characteristics of the participants (*n* = 39).

	**MCI (*****n*** **= 20)**			**mAD (*****n*** **= 19)**		
	**Control group (*n* = 9)**	**Intervention group (*n* = 11)**	***p*-value**	**Control group (*n* = 9)**	**Intervention group (*n* = 10)**	***p*-value**
Sex distribution (female/male), *n* (%)	4/5	7/4	0.653^c^	7/2	8/2	1.000^c^
Age (years)	72.0 (8.1)	70.5 (5.4)	0.617^a^	75.9 (8.0)	73.1 (5.2)	0.374^a^
Education (years)	9.6 (4.9)	9.7 (3.3)	0.766^b^	9.3 (3.0)	8.8 (3.9)	0.744^a^
**Marital status**						
Single/divorced/separated/widowed, *n* (%)	1 (11.1%)	3 (27.3%)	0.591^c^	3 (33.3%)	3 (30.0%)	1.000^c^
Married, *n* (%)	8 (88.9%)	8 (72.7%)		6 (66.7%)	7 (70.0%)	
**AD medication**						
Yes	4 (44.4%)	2 (18.8%)	0.336^c^	9 (100.0%)	10 (100.0%)	1.000^c^
No	5 (55.6%)	9 (81.2%)		0 (0.0%)	0 (0.0%)	
MMSE score	25.4 (2.4)	26.6 (3.0)	0.131^b^	19.8 (3.2)	17.8 (3.2)	0.156^b^
ADAS-cog score	12.2 (5.7)	9.8 (8.3)	0.131^b^	19.4 (9.3)	21.9 (6.0)	0.486^a^
AVLT immediate recall score	20.1 (7.0)	21.5 (5.2)	0.629^a^	12.1 (5.7)	11.8 (5.0)	0.657^a^
AVLT delay recall score	3.8 (3.7)	6.1 (3.6)	0.171^a^	2.0 (3.0)	1.0 (1.3)	0.661^b^
CDT score	13.4 (1.8)	13.3 (2.2)	0.941^b^	9.2 (3.9)	9.1 (2.6)	0.936^a^
ADCS-ADL score	57.7 (8.1)	61.7 (3.1)	0.331^b^	53.1 (8.8)	51.4 (9.7)	0.693^a^
GDS-30 score	8.0 (4.8)	5.5 (4.2)	0.152^b^	7.1 (2.3)	5.4 (3.0)	0.188^a^
PSQI score	5.9 (4.3)	6.4 (4.0)	0.801^a^	6.7 (2.9)	6.1 (3.7)	0.515^a^

a *Independent sample t-test*.

b *Mann–Whitney U-test*.

c *Fisher's exact test*.

*MCI, Mild cognitive impairment; mAD, mild Alzheimer's disease; MMSE, Mini-mental state examination; ADAS-cog, Alzheimer's Disease Assessment Scale– Cognitive Subscale; AVLT, Auditory Verbal Learning Test; CDT, Clock Drawing Test; ADCS-ADL, Alzheimer's Disease Cooperative Study–Activities of Daily Living scale; GDS-30, 30-item Geriatric Depression Scale; PSQI, Pittsburgh Sleep Quality Index*.

### Neuropsychological Tests

As shown in Tables 2, 3, after the 4-week intervention, there was a significant improvement in AVLT in T-MCI (6.36 ± 5.08 vs. −1.11 ± 4.23) and T-mAD (4.60 ± 4.77 vs. −0.11 ± 2.89) compared with the corresponding control group (*P* < 0.05). After the 12-week intervention, there was a significantly improved AVLT (9.64 ± 4.90 vs. −0.33 ± 6.10) and ADCS-ADL (3.36 ± 3.59 vs. −1.89 ± 2.71) in T-MCI, and there was a significant improvement in AVLT (5.30 ± 5.42 vs. 0.44 ± 2.40) in T-mAD compared with the corresponding control group (*p* < 0.05).

**Table 2:** Adjusted comparison of neuropsychological outcomes between changes from baseline at 4 and 12 weeks after intervention in patients with MCI (*n* = 20).

**Outcomes**	**Control group (*****n*** **= 9)**			**Intervention group (*****n*** **= 11)**			**4-week adjusted estimate (95% CI)^a^; *P*-value**	**12-week adjusted estimate (95% CI)^a^; *P*-value**
	**Baseline**	**Change from baseline at 4 weeks**	**Change from baseline at 12 weeks**	**Baseline**	**Change from baseline at 4 weeks**	**Change from baseline at 12 weeks**		
MMSE	25.44 (2.35)	0.33 (1.66)	−0.11 (1.83)	26.64 (2.98)	−0.09 (1.38)	−0.09 (1.81)	−0.7 (−2.1–0.7); 0.301	−0.02 (−1.9–1.8); 0.982
ADAS-cog	12.23 (5.71)	−1.19 (4.45)	−1.97 (3.32)	9.83 (8.30)	−2.98 (5.08)	−3.36 (5.29)	−2.0 (−6.9–2.9); 0.398	−1.3 (5.9–3.3); 0.552
AVLT immediate recall	20.11 (7.01)	−1.11 (4.23)	−0.33 (6.10)	21.45 (5.22)	6.36 (5.08)	9.64 (4.90)	7.4 (2.7–12.1); 0.004	9.6 (4.0–15.1); 0.002
AVLT delay recall	3.78 (3.67)	1.11 (3.14)	1.56 (2.24)	6.09 (3.56)	1.82 (4.19)	4.00 (3.29)	0.7 (−3.2–4.5); 0.718	2.6 (−0.3–5.6); 0.074
CDT	13.44 (1.81)	−2.00 (2.83)	−3.22 (3.42)	13.27 (2.24)	−0.36 (2.01)	−0.18 (2.56)	1.4 (−0.9–3.6); 0.222	2.5 (−0.3–5.3); 0.072
ADCS-ADL	57.67 (8.11)	−0.22 (1.92)	−1.89 (2.71)	61.73 (3.13)	0.36 (1.69)	3.36 (3.59)	0.4 (−1.3–2.1); 0.661	5.4 (2.2–8.7); 0.003
GDS-30	8.00 (4.80)	0.00 (2.45)	0.22 (2.77)	5.45 (4.16)	−0.09 (2.95)	−0.82 (3.46)	−0.03 (−2.8–2.8); 0.980	−1.1 (−4.3–2.2); 0.497
PSQI	5.89 (4.31)	0.00 (1.87)	0.44 (2.70)	6.36 (3.96)	0.64 (2.66)	−1.27 (2.76)	1.1 (−0.9–3.2); 0.266	−1.2 (−3.8–1.3); 0.319

a *Controlling for age and sex*.

*MMSE, Mini-mental state examination; ADAS-cog, Alzheimer's disease assessment scale–cognitive subscale; AVLT, auditory verbal learning test; CDT, clock drawing test; ADCS-ADL, Alzheimer's disease cooperative study–activities of daily living scale; GDS-30, 30-item geriatric depression scale; PSQI, Pittsburgh sleep quality index*.

**Table 3:** Adjusted comparison of neuropsychological outcomes between changes from baseline at 4 and 12 weeks after intervention in patients with mild AD (*n* = 19).

**Outcomes**	**Control group (*****n*** **= 9)**			**Intervention group (*****n*** **= 10)**			**4-week adjusted estimate (95% CI)^a^; *P*-value**	**12-week adjusted estimate (95% CI)^a^; *P*-value**
	**Baseline**	**Change from baseline at 4 weeks**	**Change from baseline at 12 weeks**	**Baseline**	**Change from baseline at 4 weeks**	**Change from baseline at 12 weeks**		
MMSE	19.78 (3.19)	0.89 (2.26)	0.33 (2.45)	17.80 (3.22)	0.60 (2.99)	1.40 (2.88)	−0.5 (−3.3–2.3); 0.707	1.1 (−1.7–4.0); 0.423
ADAS-cog	19.39 (9.33)	−1.07 (6.60)	−1.70 (7.51)	21.93 (6.03)	−2.44 (4.72)	−5.04 (5.17)	−0.7 (−6.3–4.9); 0.786	−3.3 (−10.1–3.5); 0.319
AVLT immediate recall	12.11 (5.73)	−0.11 (2.89)	0.44 (2.40)	11.83 (4.99)	4.60 (4.77)	5.30 (5.42)	4.3 (0.3–8.4); 0.039	4.9 (0.4–9.4); 0.035
AVLT delay recall	2.00 (2.96)	0.67 (1.41)	1.00 (2.18)	1.00 (1.33)	0.00 (1.49)	1.80 (2.57)	−0.7 (−2.2–0.8); 0.345	1.0 (−1.3–3.3); 0.383
CDT	9.22 (3.90)	0.22 (3.07)	−0.67 (3.84)	9.10 (2.56)	1.60 (3.34)	1.00 (2.94)	0.8 (−2.4–3.9); 0.601	1.2 (−2.3–4.6); 0.479
ADCS-ADL	53.11 (8.81)	−0.11 (3.72)	2.33 (6.24)	51.40 (9.67)	0.10 (6.40)	2.10 (8.60)	0.1 (−5.2–5.4); 0.968	−0.8 (−8.1–6.5); 0.817
GDS-30	7.11 (2.32)	1.44 (4.16)	2.33 (6.38)	5.40 (3.03)	0.10 (2.69)	−0.70 (2.40)	−1.8 (−5.4–1.7); 0.280	−3.4 (−8.4–1.5); 0.158
PSQI	6.73 (2.85)	1.33 (2.00)	1.56 (3.24)	6.10 (3.67)	−0.2 (2.57)	−0.4 (2.12)	−1.8 (−4.1–0.5); 0.108	−1.9 (−4.7–0.8); 0.158

a *Controlling for age and sex*.

*MMSE, mini-mental state examination; ADAS-cog, Alzheimer's disease assessment scale–cognitive subscale; AVLT, auditory verbal learning test; CDT, clock drawing test; ADCS-ADL, Alzheimer's disease cooperative study–activities of daily living scale; GDS-30, 30-item geriatric depression scale; PSQI, Pittsburgh sleep quality index*.

### Neurobiological Tests

As shown in Tables 4, 5, most inflammatory markers remained unchanged after sport stacking. After the 12-week intervention, there was a significant improvement in BDNF in T-MCI (41.6 ± 24.3 vs. −7.5 ± 55.2 ng/ml) and T-mAD (29.9 ± 33.4 vs. −23.5 ± 25.5 ng/ml), as compared with the corresponding control group (*p* < 0.05). We found a significantly increased IGF-1 (2.7 ± 5.3 vs. −4.7 ± 11.6 ng/ml) and IL-6 (0.5 ± 0.2 vs. 1.0 ± 0.2 pg/ml) in T-MCI compared with the control group (*p* < 0.05).

**Table 4:** Adjusted comparison of neurobiological measurements between changes from baseline at 12 weeks after intervention in patients with MCI (*n* = 16).

	**Control group (*****n*** **= 6)**		**Intervention group (*****n*** **= 10)**		**Between-group difference (95%CI)^a^**	***P*-value**
	**Baseline**	**Change from baseline at 12 weeks**	**Baseline**	**Change from baseline at 12 weeks**		
Aβ-40 (pg/ml)	263.9 (71.0)	−44.8 (23.0)	247.3 (57.2)	−69.1 (39.6)	−18.7 (−56.8, 19.4)	0.305
Aβ-42 (pg/ml)	61.9 (27.2)	3.5 (48.7)	44.2 (6.9)	8.6 (16.6)	12.6 (−21.4, 46.6)	0.434
Aβ42/Aβ40	0.23 (0.08)	0.1 (0.2)	0.19 (0.06)	0.1 (0.1)	0.1 (−0.1, 0.3)	0.470
BDNF (ng/ml)	159.7 (65.9)	−7.5 (55.2)	110.9 (39.3)	41.6 (24.3)	56.6 (12.8, 100.4)	0.016
IGF-1 (ng/ml)	17.2 (9.2)	−4.7 (11.6)	10.1 (2.1)	2.7 (5.3)	9.4 (0.5, 18.2)	0.040
TNF-a (pg/ml)	3.7 (1.1)	1.1 (0.5)	4.0 (1.3)	0.7 (0.8)	−0.3 (−1.1, 0.5)	0.405
IL-6 (pg/ml)	2.1 (0.3)	1.0 (0.2)	2.4 (0.3)	0.5 (0.2)	−0.4 (−0.6, −0.2)	0.001
sTREM-2 (pg/ml)	19.4 (13.3)	−4.9 (18.6)	11.3 (2.9)	0.1 (6.6)	7.9 (−5.4, 21.2)	0.220

a *Controlling for age and sex*.

*Aβ-40, amyloid β-protein-40; BDNF, brain-derived neurotrophic factor; IGF-1, insulin-like growth factor-1; TNF-α, tumor necrosis factor-alpha; IL-6, Interleukin-6; sTREM2, soluble trigger receptor expressed on myeloid cells 2*.

**Table 5:** Adjusted comparison of neurobiological measurements between changes from baseline at 12 weeks after intervention in patients with mild AD (*n* = 16).

	**Control group (*****n*** **= 7)**		**Intervention group (*****n*** **= 9)**		**Between-group difference (95%CI)^a^**	***P*-value**
	**Baseline**	**Change from baseline at 12 weeks**	**Baseline**	**Change from baseline at 12 weeks**		
Aβ-40 (pg/ml)	212.0 (55.9)	−29.0 (43.5)	219.4 (49.1)	−25.2 (35.7)	−1.2 (−47.0, 44.6)	0.954
Aβ-42 (pg/ml)	40.6 (5.2)	5.9 (10.5)	57.9 (30.3)	−3.8 (31.0)	−8.9 (−38.9, 21.2)	0.531
Aβ42/Aβ40	0.20 (0.02)	0.07 (0.09)	0.26 (0.10)	0.03 (0.16)	−0.03 (−0.2, 0.1)	0.696
BDNF (ng/ml)	137.9 (29.1)	−23.5 (25.5)	120.1 (25.8)	29.9 (33.4)	50.5 (13.9, 87.0)	0.011
IGF-1 (ng/ml)	10.0 (1.6)	−0.03 (3.32)	14.2 (8.2)	−1.5 (8.5)	−1.2 (−9.3,6.9)	0.752
TNF-a (pg/ml)	3.8 (1.1)	0.8 (0.3)	3.8 (0.6)	0.3 (0.4)	−0.4 (−0.9, 0.02)	0.060
IL-6 (pg/ml)	2.3 (0.7)	1.2 (0.7)	2.4 (0.1)	0.7 (0.7)	−0.5 (−1.3, 0.4)	0.243
sTREM-2 (pg/ml)	9.7 (1.9)	0.2 (4.6)	18.0 (12.7)	−6.1 (13.0)	−6.4 (−18.9, 6.2)	0.291

a *Controlling for age and sex*.

*Aβ-40, amyloid β-protein-40; BDNF, brain-derived neurotrophic factor; IGF-1, insulin-like growth factor-1; TNF-α, tumor necrosis factor-alpha; IL-6, Interleukin-6; sTREM2, soluble trigger receptor expressed on myeloid cells 2*.

### Inter-cohort fNIRS Analysis

**Figure 3:**
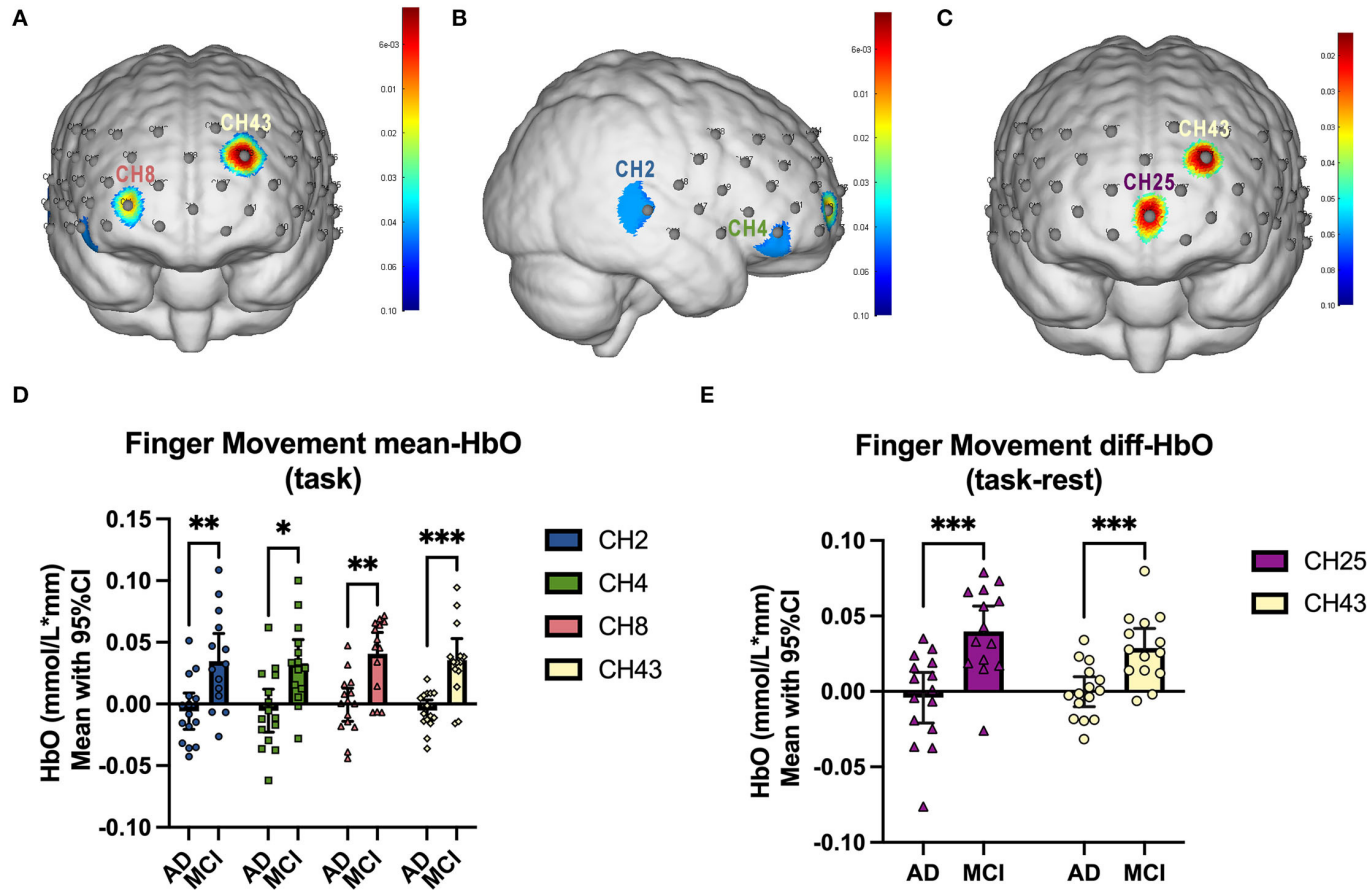
fNIRS comparison between mAD and MCI subjects. **(A,B)** The front and right lateral view of significant channels in mean Oxy-Hb comparison, and the statistics were presented in **(D)**. **(C)** The front view of significant channels in diff-Oxy-Hb (task-rest) comparison, and the statistics were presented in **(E)**. *p < 0.05; **p < 0.01; ***p < 0.001.

We firstly compared the mean Oxy-Hb concentration during the SFS task between mild AD cohort and MCI cohort, as well as the Oxy-Hb change between task and baseline. As is presented in Figures 3A,B,D, compared to MCI patients, significant lower Oxy-Hb concentrations during the task were exhibited in mild AD patients in channel 2, 4, 8, and 43 [mean with 95% CI, mAD vs. MCI, CH2: −0.0058 (−0.0205, 0.0088) vs. 0.0348 (0.0122, 0.0573), *p* = 0.006; CH4: −0.0054 (−0.0229, 0.01200) vs. 0.0329 (0.0137, 0.0521), *p* = 0.019; CH8: −0.0006 (−0.0142, 0.0129) vs. 0.0408 (0.0235, 0.0581), *p* = 0.008; CH43: −0.0053 (−0.0137, 0.0032) vs. 0.0354 (0.0178, 0.0530), *p* = 0.0004; all after FDR corrected]. Next, the Oxy-Hb change (task-rest) during SFS task in mild AD and MCI cohorts were shown in Figures 3C,E. Compared with MCI subjects, mild AD patients showed significant lower differences of Oxy-Hb level between the task and rest in channel 25 and channel 43 [mean with 95% CI, mAD vs. MCI, CH25: −0.0042 (−0.0208, 0.0126) vs. 0.0397 (0.0228, 0.0567), *p* = 0.0005; CH43: −0.0001 (−0.0101, 0.0098) vs. 0.0287 (0.0156, 0.0418), *p* = 0.0007; all after FDR corrected].

### Functional Connectivity Change After Intervention

**Figure 4:**
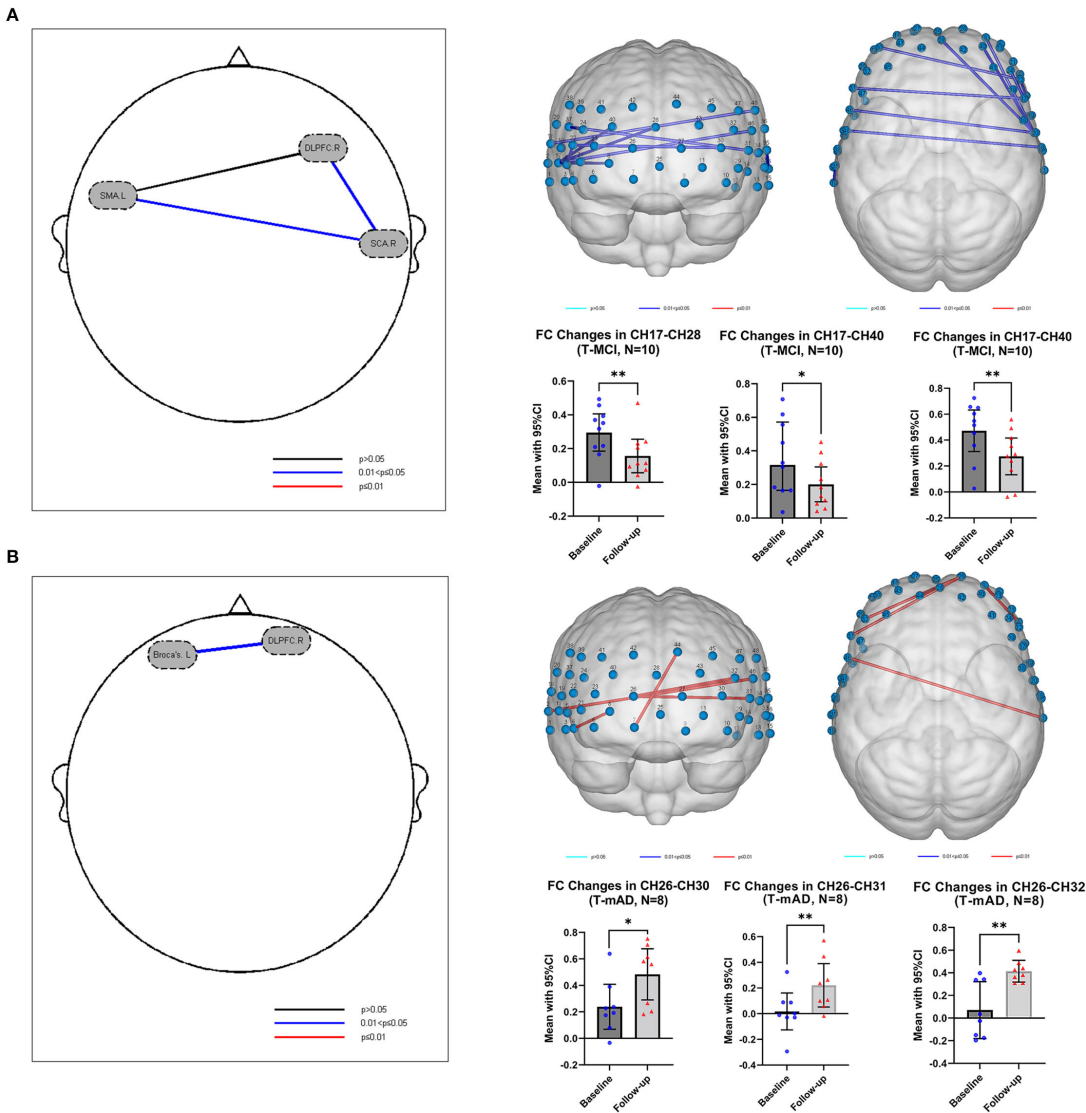
Functional connectivity change after intervention. **(A)** The functional connectivity change in T-MCI (*n* = 10) after sport stacking training for 12 weeks. **(B)** The functional connectivity change in T-mAD (*n* = 8) after sport stacking training for 12 weeks. Each dot represents the FC value of each channel in T-MCI and T-mAD participants at baseline and at follow-up. *p < 0.05. No significant differences between intervention groups and control groups were found. **p < 0.01.

In both T-MCI and T-mAD groups, the changes of R-values among every two ROIs for each subject between baseline and 12-week follow-up were compared by paired *t*-test and were corrected by FDR. As is shown in Figure 4A, compared to baseline, patients with MCI (*n* = 10) showed significant decrease of FC between SCA.R and SMA.L (mean of R, baseline vs. follow-up: 0.5040 vs. 0.2744, *p* = 0.038, FDR corrected), and between SCA.R and DLPFC.R (mean of R, baseline vs. follow-up: 0.3454 vs. 0.1786, *p* = 0.038, FDR corrected) after sport stacking training for 12 weeks. As for T-mAD group (n=8), a significant increase of FC was analyzed between DLPFC.R and Broca's.L after sport stacking intervention (mean of R, baseline vs. follow-up: 0.1085 vs. 0.3727, *p* = 0.024, FDR corrected) (Figure 4B).

### Change of Brain Activation After Intervention

**Figure 5:**
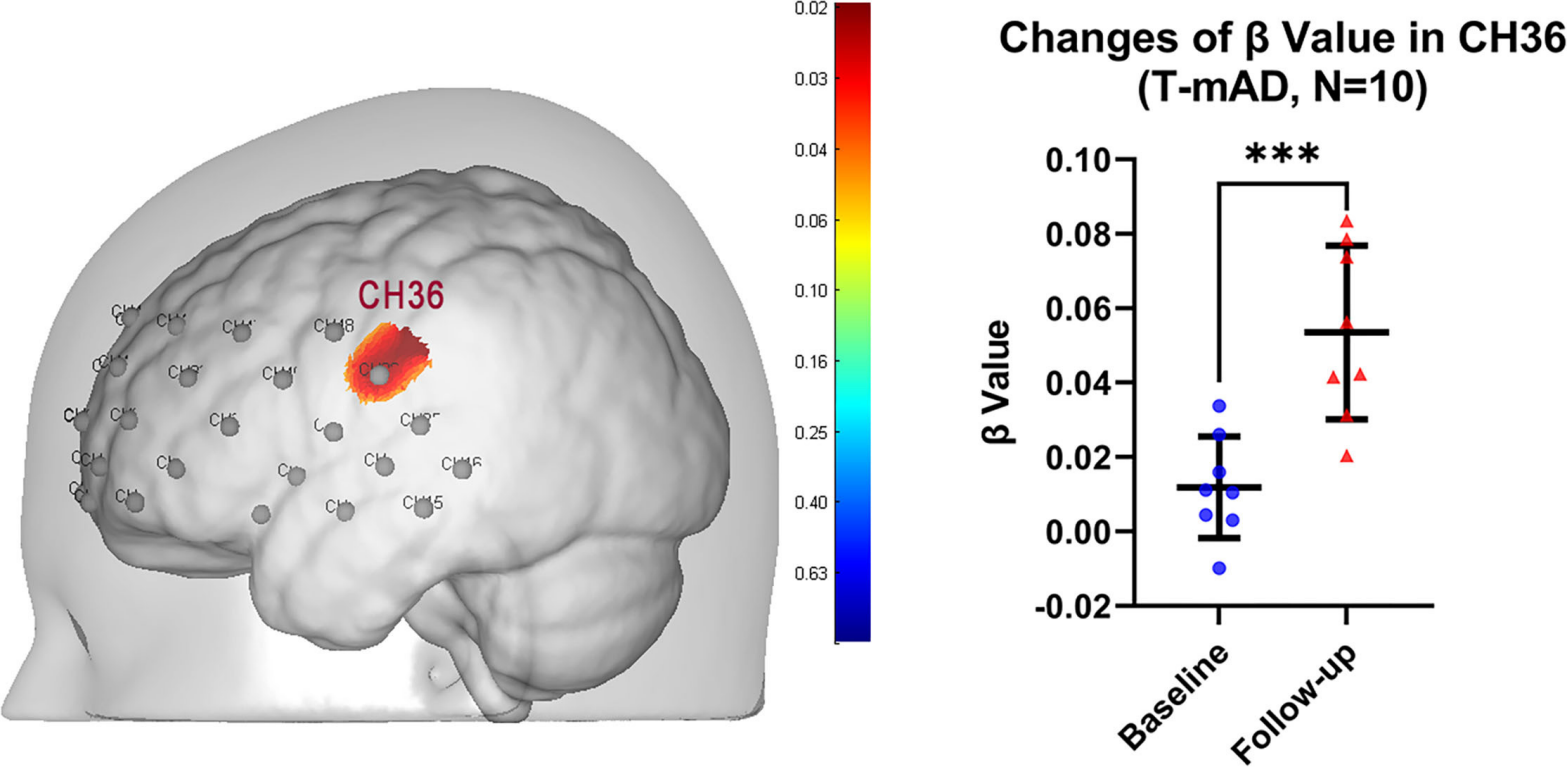
Change of brain activation after intervention in T-mAD subjects. CH36 area was significantly activated after sport stacking intervention (left) and statistical results (right). ***p < 0.001. Each dot represents the individual β value of T-mAD participants in CH36 at baseline and at follow-up. No significant brain activation differences in T-MCI subjects were found, and no significant differences between intervention groups and control groups were found.

In SFS task fNIRS analysis, the β value derived from GLM from each channel represents cortical activation. We compared the β value of each channel for each T-MCI, T-mAD, C-MCI and C-mAD subjects between baseline and 12-weeek follow-up. We only found that in T-mAD group, CH36 area was significantly activated after sport stacking intervention, which overlaps cortex of left supramarginal gyrus (SMG.L) [mean with 95% CI of β, baseline vs. follow-up: 0.0119 (0.0004, 0.02329) vs. 0.0535 (0.03401, 0.0730), *p* = 0.0003, FDR corrected] (Figure 5), however, no significant differences between baseline and follow-up were found in the other three groups. Further, we correlated the changes of β value in CH36 of T-mAD subjects with their improved performance of ALVT (difference value) and changes of BDNF levels by Pearson Correlation analysis, and we found that the change of β value in CH36 was correlated with BDNF levels (coefficient value *r* = −0.780, *p* = 0.039) and with changes of AVLT scores (*r* = −0.875, *p* = 0.004), and AVLT performance was also correlated with increasement of BDNF levels (*r* = 0.763, *p* = 0.046). No significant differences were found in T-MCI, C-MCI and C-mAD groups, and no significant differences were found between T-MCI and C-MCI groups, and between T-mAD and C-mAD groups at follow-up.

## Discussion

The present study was aimed at investigating the effects of sport stacking on cognitive performances in individuals with mild AD and MCI. There was apparent evidence of improved cognitive function in tests of AVLT and ADCS-ADL. Our results showed that the intervention effectively improved episodic memory of patients with mild AD and MCI and improved the activities of living of patients with MCI. Moreover, this 12-week sport stacking added to usual care successfully increased the expression of some neuroprotective growth factors, including BDNF in both mild AD and MCI patients and IGF-1 in MCI subjects. More importantly, we found the functional connectivity in MCI patients between DLPFC.R and SCA.R as well as between SMA.L and SCA.R decreased after training. In contrast, in mild AD patients, the brain regional function connection was increased between DLPFC.R and Broca's L. In addition, the activation of channel 36, which was located in the left primary somatosensory cortex, was significantly increased after 12-week of sport stacking, and this increased activation was correlated with the improved cognitive function (AVLT) as well as the increased level of BDNF. These findings support the hypothesis that sport stacking would improve the cognitive function of patients with mild AD and MCI and that sport stacking would have an upregulating effect on anti-inflammatory cytokines and neuronal plasticity.

Sport stacking, which combines game and exercise, is a new sport. Our study indicated that a significant increase in the score of AVLT was measured in the sport stacking group after 12 weeks which means our training could improve all patients' episodic memory. Our results were consistent with that reported in a randomized controlled study of Hagovska and Nagyova (2017), which showed that cognitive training combined with physical training could significantly improve AVLT scores, indicating greater improvement of the memory in older people with mild cognitive impairment. A systematic study of Law et al. (2014) presented similar results in the improvement of general cognitive functions and memory in older adults after the intervention of combined exercise and cognitive training. Similar studies in the literature also found improvements in the previously mentioned cognitive domains (Phirom et al., 2020). In addition, there is growing evidence that the combination of physical and cognitive activities may have synergistic effects (Kraft, 2012; Gheysen et al., 2018). Although physical exercise contributes to plasticity, cognitive activities lead to changes in plasticity (Fissler et al., 2013). This combined benefit from exercise and cognitive stimulation would be consistent with previous animal research, which showed that this cognitive benefit had been found to be from different mechanisms (cell proliferation and cell survival, respectively, Olson et al., 2006; Fabel et al., 2009). And this combined-effect hypothesis indicated that simultaneous exercise and cognitive interventions could further increase cognitive benefits. Similarly, in line with previous studies, these findings showed that when physical exercise was cognitively challenging, the cognitive benefits were greater than traditional exercise (Anderson-Hanley et al., 2012).

Besides improvements in cognitive performances, activities of living of patients with MCI, as assessed with the ADCS-ADL, ameliorated. Sport stacking is a coordinated exercise of hands and eyes. Hand movement can improve hand function, stimulate brain function, reduce the occurrence and development of brain-related diseases (Geng, 2012), and enhance the learning ability of students with intellectual disabilities (Qu et al., 2012). Nyberg et al. found that finger tapping can stimulate the motor cortex in the brain (Nyberg et al., 2006). Finger exercise can also maintain or improve the ability of daily living and self-care and handling tools in patients with dementia (Zhang et al., 2010). Wang and Kui (2014) also suggested that finger movement could improve ADL by promoting blood circulation in the brain and the central nervous system, thereby improving brain function.

However, we did not find significant mAD results in the intervention group for ADL and other cognitive domains, probably due to the short duration of our intervention, while the overall score of patients with mild AD is changing in the direction of improvement. There is evidence that the severity of neurocognitive impairment can regulate the cognitive effect of combined cognitive and sports training (Bamidis et al., 2015). The increase in the severity of neurocognitive impairment may decrease the effect of the intervention (Bamidis et al., 2015). This can be explained by a reduction in the structural brain capacity of participants with more severe neurocognitive impairment (e.g., reduced number of neurons and synapses), which may result in limited resources for training-induced benefits (Bamidis et al., 2015). As a result, it may be more difficult to induce cognitive benefits in people with dementia than in people with MCI or healthy older people.

Our results indicate that the concentration of BDNF in peripheral blood increased significantly in all patients who participated in 12-week sport stacking, and the concentration of IGF-1 in peripheral blood only increased significantly in patients with MCI. Consistent with the results of our study, previous research has suggested that multicomponent exercise could increase BDNF concentrations (Wang et al., 2020), and physical exercise has been shown to increase IGF-1 levels in patients with MCI (Baker et al., 2010). Exercise-related upregulation of BDNF and IGF-1 may help to offset the age-related decline in synaptogenesis, neurogenesis, angiogenesis, synaptic plasticity, and learning and memory, thus making the brain more flexible in dealing with age-related structural and functional neurodegeneration (Cotman and Berchtold, 2002, 2007). Together, these findings suggest that the production of BDNF and IGF-1 may be a mechanism responsible for cognitive improvement after sport stacking. In addition, our results show that non-strenuous exercise games such as sport stacking can improve BDNF and IGF-1 levels, which is of great significance for the exercise program of the elderly because the health status of the elderly is often unable to do strenuous exercise and hard to stick to.

Regarding IL-6, our results indicate that the change of IL-6 concentration in blood serum increased in both intervention groups, whereas only changes from the MCI group became statistically significant. Consistent with the results of our study, Behrendt et al. showed that both open and closed skill exercises were equally efficient in acutely increasing the IL-6 serum levels in healthy older adults (Behrendt et al., 2021). However, Forti et al. (2014) showed results, contrary to ours, that IL-6 levels significantly decreased after 12 weeks of strength training in 20 older adults. It is reported that peripheral IL-6 concentration increases sharply during physical exercise and returns to the baseline level within 24 h (Fischer, 2006). In this case, IL-6 is considered to have anti-inflammatory effects and may be a factor in reducing the risk of chronic inflammation and neurological diseases by exercising regularly (Funk et al., 2011; Smart et al., 2011). As IL-6 plays multiple roles in a variety of biological processes of the human body (Norman et al., 2018; Ellingsgaard et al., 2019), further research is needed to better understand the relationship between IL-6 and exercise and its impact on neurocognitive processes.

In this study, the differences of mean and difference of Oxy-Hb concentration between mild AD and MCI subjects indicated that the cortical blood flow and neuronal activity in mAD patients were significantly reduced, consistent with previous studies (Herrmann et al., 2008; Haberstumpf et al., 2022). One of the important findings of this study is the different outcomes of FC analysis in T-mAD and T-MCI during resting state, compared with baseline and 12-week follow-up. There was a decrease of FC between DLPFC.R and other ROIs in MCI, while an increase was displayed in subjects with mild AD. The DLPFC is an associative cortical region that is often described as a functional hub enabling a host of higher-order processes, including working memory (McKendrick et al., 2014), mentalizing, attention, and response inhibition (Rodrigo et al., 2014). In functional studies of healthy participants, faster processing speeds have been related to reduced directed functional connectivity and activation of the DLPFC (Motes et al., 2018). Based on these studies and our results, we speculated that the decrease of FC in between ROIs in MCI patients may correlate with the neuronal plasticity changes after sport stacking. Although DLPFC activation is commonly observed to increase in a parametric manner with workload (Ayaz et al., 2012), increased DLPFC activation may also occur as a compensatory mechanism to reductions in available neural resources (Stuss and Knight, 2002) or alternatively, inefficient utilization of neural resources (Neubauer and Fink, 2009). which may indicate that patients with MCI may have higher FC values between these ROIs at baseline because of functional compensation in these brain areas, but after sport stacking intervention, the neural remodeling, while improving cognitive function, also regulated the compensation of brain regions and thus reduced FC between ROIs were found. Our study also found the increased activation in CH36, overlapping the cortex of SMG.L, in mild AD subjects under the SFS task and was correlated with increased cognitive performance and upregulated level of BDNF. A previous study has demonstrated that SMG.L is involved in goal orientated cognition (Smallwood et al., 2021). This may prove that sport stacking may arouse a multi-system effect to improve cognition through enhancing neuronal plasticity and boosting the production of BDNF.

The present study has some limitations. Firstly, the sample size in our study is small and it might lead to statistical errors. In order to explore the further impact of sport stacking on the elderly with dementia and avoid statistical errors in the process of data analysis, future studies should expand the sample size; secondly, the period of our intervention was only 12 weeks, and we did not see any significant improvement in outcome indicators other than episodic memory in all patients, probably because the intervention time was relatively short; thirdly, although sport stacking was confirmed to be effective for individuals with MCI and AD, larger trials comparing sport stacking with other active control interventions such as aerobic exercise need to confirm or refute our findings. Finally, our results are restricted to patients with mild AD and MCI and cannot be generalized to those with more dementia types.

Although our study had these limitations, results still suggested the effectiveness of sport stacking and its benefits on participants' memory and activities of daily living, possibly via upregulation of BDNF and IGF-1. What's more, our study provides a new method for non-pharmaceutical treatment for patients with early stages of cognitive impairment.

## Conclusion

Our findings suggested that sport stacking is effective for patients with MCI and mild AD, possibly through increasing the expression of neuroprotective growth factors and enhancing neural plasticity to improve neurocognitive performance.

## Data Availability Statement

The raw data supporting the conclusions of this article will be made available by the authors, without undue reservation.

## Ethics Statement

The studies involving human participants were reviewed and approved by Ethics Committee of the First Affiliated Hospital of Chongqing Medical University. The patients/participants provided their written informed consent to participate in this study.

## Author Contributions

FD, YLü, ZY, WZ, YT, JL, DL, JY, and HJ contributed to study design, implementation, and interpretation. ZY, WZ, JS, YLi, and XL contributed to the management of data. ZY, DL, and WZ contributed to the analysis and interpretation of neuropsychological data. ZY, S-sZ, and YT contributed to biomarker data analysis and interpretation. WZ contributed to fNIRS data analysis and interpretation. ZY and WZ contributed to the drafting of the manuscript. FD, YLü, ZY, and WZ contributed to the critical revision of the manuscript. All the authors contributed to the data collection. All authors contributed to the article and approved the submitted version.

## Funding

This work was supported by the Intelligent Medicine Project of Chongqing Medical University (Grant No. ZHYX2019003) and the Nursing Research Fund Project of the First Affiliated Hospital of Chongqing Medical University (Grant No. HLJJ2015-06 and HLJJ2020-06). This study was supported by the Geriatric Memory Clinic at the First Affiliated Hospital of Chongqing Medical University, the Department of Histology and Embryology and Laboratory of Stem Cell and Tissue Engineering in Chongqing Medical University, Chongqing, China.

## Conflict of Interest

The authors declare that the research was conducted in the absence of any commercial or financial relationships that could be construed as a potential conflict of interest.

## Publisher's Note

All claims expressed in this article are solely those of the authors and do not necessarily represent those of their affiliated organizations, or those of the publisher, the editors and the reviewers. Any product that may be evaluated in this article, or claim that may be made by its manufacturer, is not guaranteed or endorsed by the publisher.
