# Supplementary material for: Effects of Sport Stacking on Neuropsychological, Neurobiological, and Brain Function Performances in Patients With Mild Alzheimer's Disease and Mild Cognitive Impairment: A Randomized Controlled Trial
Source: Front Aging Neurosci. 2022 May 12;14:910261. doi: 10.3389/fnagi.2022.910261 (PMC9133718; doi:10.3389/fnagi.2022.910261)
Supplement: Supplementary file 1 [file Data_Sheet_1.docx]

Supplementary Material

# Supplementary Figure 1

**
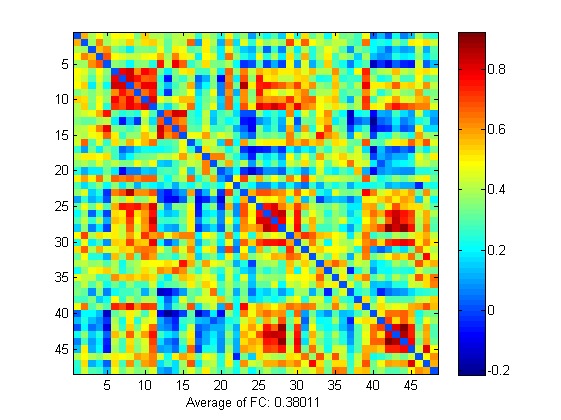
**

**Supplementary Figure 1.** Correlation matrix of 48 × 48 channels.
